# Medical and Health Care Professionals’ Sexuality Education: State of the Art and Recommendations

**DOI:** 10.3390/ijerph17072186

**Published:** 2020-03-25

**Authors:** Valeria Verrastro, Valeria Saladino, Filippo Petruccelli, Stefano Eleuteri

**Affiliations:** 1 Faculty of Medical and Surgical Sciences, University “Magna Graecia” of Catanzaro, Via Scesa Eroi, 23, 88100 Catanzaro, Italy; valeriaverrastro@unicz.it; 2 Faculty of Economics, Mercatorum University, Piazza Mattei, 10, 00186 Rome, Italy; valeriasaladino26@gmail.com (V.S.); filippopetruccelli@gmail.com (F.P.); 3 Faculty of Medicine and Psychology, Sapienza University of Rome, Via dei Marsi 78, 00178 Rome, Italy

**Keywords:** education, health care professionals, sexuality, training

## Abstract

Sexuality is considered an important aspect of holistic care, but research has shown that it is often not considered, as it should be, in health services. Addressing clients’ sexuality requires a multidisciplinary approach and is not the responsibility of a single professional. The literature underlines that university students or those working in hospitals and other health care facilities are not adequately prepared to meet patients’ needs regarding sexuality. The objective of this study was, therefore, to review the scientific literature addressing training courses for health professionals in sexuality between 2000 and 2020. Several studies have shown enhancement in health care professionals’ ability to deal with patients’ sexuality issues after participating in sexuality education programs, regardless of the course load and modality, even if the long-term effects have still to be proved. Health care professionals therefore require education in the area of sexuality, regardless of their discipline. According to the articles reviewed, in order to improve the performance and comfort level of health care professionals to deal with patients’ sexuality, investments in training are necessary. Further evaluations of interdisciplinary sexuality education programmes should use larger samples and explore the differences across disciplines.

## 1. Introduction

Sexuality is considered an important aspect of holistic care, but research has shown that it is often not considered, as it should be, in health services. Addressing clients’ sexuality requires a multidisciplinary approach and is not the responsibility of a single professional [1]. Sexuality has been defined as the way people experience themselves and others as sexual beings, including sexual activity, sexual orientation, gender identity and gender roles, eroticism, pleasure, intimacy, and reproduction [2]. It represents a need in everyone’s life, a fundamental and natural element, regardless of age or physical state. The expression of sexuality is an integral part of every person, it is a basic human right, and continues throughout life [3]. 

Most health professionals (HCP) do not proactively discuss sexuality issues with service users—recent research in the UK [4] has shown that although 60% of HCP agreed that sexual issues should be addressed, only 6% started frequent discussions with patients themselves due to personal blockages, like lack of training (79%), lack of time (67%), and embarrassment (50%). It is, therefore, important to develop potential strategies to overcome these barriers, such as training, policy development, availability of written information for service users, and communication between professionals. However, limitations of their implementation, such as structural, organizational, and personal factors, should be considered. 

There are some diseases, such as diabetes, that can cause reproductive and sexual health problems. Health professionals should be aware of the prevention and early diagnosis of reproductive and sexual health (RSH) problems in patients with chronic condition. Most health care professionals do not have an in-depth knowledge of the effects of pathology on sexual health, the most frequent RSH problems in patients, and the side effects of drugs used to treat RSH. The initial and basic rule for competent RSH counseling is to follow appropriate training [5].

Sexuality education is a method to reduce common negative stereotypes about this aspect of life [6] and it is not yet widespread in medical practice to investigate and treat sexual dysfunction. A rational approach to the problem should involve in-depth discussion and investigation of the root causes. Knowledge and attitudes towards sexuality are, therefore, particularly important in those who educate health care professionals. In particular, medical doctors are reluctant to ask patients details on their sexual life and sexual history. This may be a reluctance inherited from the lack of training during years of medical education, where the topic of sexual medicine is rather dormant and sexuality quite unheard of. This discomfort is easily felt by patients and establishes a vicious circle, or an attitude of “we don’t ask and they don’t tell” [6]. Taking into consideration more specific aspects, health care providers may hold biases towards LGBTQ+ persons and may not be educated on their needs [7,8].

Increasing knowledge and awareness about this topic can contribute to minimizing the provision of inefficient care or unsatisfactory professional performance concerning sexual education or detection or prevention of problems. Therefore, higher-education institutions should provide thorough training for their students to effectively deal with this issue, and hospitals and other health care facilities should also provide proper training for their health professionals [9,10]. 

Some researchers have emphasised the cultural aspects of addressing sexual matters with patients, reporting that talking about this subject in certain cultures might be more difficult than in others [11,12]. Haboubi and Lincoln [4] performed a large survey (*N* = 813) including British nurses, doctors, physiotherapists, and occupational therapists. They found that both physiotherapists and occupational therapists had received less training on, lower comfort levels with, and were less willing to discuss sexual issues than doctors and nurses, while doctors discussed sexual issues significantly more often than others.

### Aim of the Study

The aim of the present study was to conduct a literature review on medical and health care professionals’ sexuality education. 

## 2. Materials and Methods

We conducted a review of the literature on and related to medical and health care professionals’ sexuality education. Electronic databases utilized included: Opendissertation, Communication and Mass Media Complete, Education Source, Old Testament Abstract, Psychology and Behavioural Sciences Collection, PsycINFO, SocINDEX with full text, and the Atla Religion Database with AtlaSerials PLUS.

### 2.1. Inclusion Criteria

We utilized the following search terms: “sex” (OR “sexuality” OR “sexual health”) AND training (OR “education interventions” OR “education”) AND medical doctors (OR “health care professionals”). Of the 539 articles returned from the search, eight were retained for the current review after screening their titles and abstracts. The references of these eight articles were also searched, of which three articles were deemed relevant and were included, allowing us to review 11 articles in total (articles included in the review are shown in Table 1). The inclusion criteria were as follows: (1) articles on training, and education regarding sexuality; (2) original articles written in English; (3) articles published in peer-reviewed journals between 2000 and 2020; (4) articles reporting empirical results on the intervention; (5) articles involving HCPs or students in degree courses for HCPs. 

### 2.2. Exclusion Criteria

Articles published in a language other than English, duplicate articles (primary articles were retained), and irrelevant articles (determined after looking through the abstracts or text) were excluded from the review pool.

## 3. Results

All studies analyzed showed that training is crucial for efficient and effective communication on sexuality. However, training was quite varied based on the duration (we have seen that the training duration can go from 40 minutes to two years) and on the content (more lecture-based, or more interactive).

In a study conducted 20 years ago [13], a majority of practice nurses (62%) reported having undertaken at least one course dealing with sexual health issues in the last five years. Younger nurses were significantly more likely to have reported training, as were nurses working more hours. Practice nurses who reported having received training in sexual health in the last five years had significantly lower mean scores on statements assessing positive attitudes towards discussing sexual health issues with patients when compared with practice nurses who reported no training. Practice nurses who reported having received training in sexual health in the last five years also had significantly higher mean scores on statements assessing negative attitudes towards discussing sexual health with patients (with the exception of male patients) when compared with practice nurses who reported no training. These findings suggest that practice nurses who report training in sexual health hold more positive attitudes towards discussing sexual health issues with patients.

A comprehensive long-term educational intervention program was developed with the aim of helping nurses and physicians to talk about sexual issues with their patients, focusing on cancer and its treatment [23]. The training was made by two workshops of five hours: one focused on oncology and sexual health care, addressing attitudes and practices. Various teaching methods were utilized, such as short lectures, group discussions, and case studies in pairs where participants practised starting discussions about sexuality and taking a short sexual history. The second workshop, with the same participants, was held a year later. It focused on building upon the contents of the baseline workshop, and by including more role play exercises designed to practice communication about sexuality-related issues. Moreover, educational meetings among staff and change agents on wards were held: they were short staff meetings (max. 20–30 min in duration) on each unit following the workshops. These meetings were intended to follow-up on the training and highlight the various steps in the project. Different issues were discussed, such as specific communication strategies, practical issues in providing sexual health care, and screening possibilities. A staff pocket guide was developed for nurses and physicians as an aid to initiate communication with patients about sexual-related issues and to do a basic assessment. This study was a pre- and post-test time-series design with a baseline measure before the start of the intervention at Time 1 (T1), 10 months later at Time 2 (T2), and at 16 months at Time 3 (T3). Those who had attended the workshops reported initiating the discussion more often than those who did not attend, both in 2011 (78% vs. 49%, *p* < 0.05) and 2012 (91% vs. 46%, *p* < 0.005). Furthermore, those who reported to have had some sexual health care education/courses in the last 5 years were more frequent initiators than those who did not have further education (71% vs. 48%, *p* < 0.001). Significant changes (*p* < 0.038) in the frequency of perceived barriers were found between T1–T3, such that the barrier of lack of training decreased by 22%. All of the respondents who had attended the workshops rated them as very useful/useful. Overall, the website was considered very useful/useful by 89%, and the pocket guide was considered very useful/useful by 73%. 

A quasi-experimental design was used to evaluate the effectiveness of the sexual health care education on nursing students’ knowledge, attitude, and self-efficacy related to sexual health care [20]. The experimental group (95 subjects) received a 12-week program, but the control group (95 subjects) did not. The education program was designed to include three subjects. The first subject was to introduce the bio-psycho-social aspects of sexuality and nurses’ roles in promoting and maintaining sexual health in order to clarify current personal beliefs, values, experiences, their own sexual identity, other personal views, and problems relating to nursing and sexuality. The second subject focused on the bio-psycho-social effects of illness, disability, and medical treatment to sexual problems in order to build students’ knowledge on sexual functions. The last one aimed to develop practice skills related to assessment and communication about sexual health care in order to assist the patient/client to maintain or attain sexual health. Different interventions were used: teacher instruction, group discussion, role-playing exercises, case analysis, value clarification, brain-storming, modeling, testing, visual media, pictures, handouts, and a reflection report. The sexual health history report was used to illustrate and reinforce knowledge, increase tolerance to sexual differences, decrease judgmental attitudes, and further improve self-efficacy and build confidence in discussing sexual concerns with patients.

There were a total of 12 sessions (one session per week with a duration of 100 minutes) in the education program. This study demonstrated that sexual health care education can help nursing students enhance their knowledge and explore their own values, as well as feelings on patient sexuality, and play an important role in preparing students to meet future challenges related to sexual health. The results of this research clearly demonstrate that a well-designed sexual health care education program can significantly improve nursing students’ knowledge levels of sexual healthcare, enhance positive changes of their attitudes about sexual healthcare, and increase their confidence in discussing sexual concerns with patients in the future.

In a New Zealand study [15], workshop participants showed significant enhancement in knowledge, skills, and comfort after the education session. The workshop lasted two days. A number of these outcomes were maintained at the six-month follow-up. There was an increase in the level of reported activity in addressing patient sexual health in the six months to follow-up, compared to a similar time period preceding the workshop. In contrast, the control group had similar pre-workshop scores to the workshop participants, but recorded no differences at the follow-up. The teaching methodology was based on multimodal approaches, incorporating lectures, didactic input observation of a role play, small-group brain-storming, and discussions based on patients’ sexual problems.

A study in the Netherlands [16] carried out training for physicians, psychologists, and social workers with three units of three hours each, and for other disciplines with two units of three hours each. For each discipline, the training consisted of knowledge transfer, exercises on actively ‘‘talking sex’’, and role-playing exercises with the cooperation of volunteer rehabilitation patients and a training actress, and a range of discipline-adapted explicit case histories were provided to motivate participants with an appeal on professional pride: ‘‘What can your profession offer in the area of sexuality and sexual problems that cannot be offered by other disciplines?”.

Information was given on sexuality, on treatment aspects, on sexual and intimacy effects of the most common diagnostic groups, ideas of professionals from various disciplines on dealing with sexuality, and talking about sex. In the interval(s) between the sessions, the participants had to do practical homework. This consisted of ‘‘talking sex’’ with their own rehabilitation patients in an appropriate manner, fitting to their own discipline. All disciplines showed significantly higher scores after training, but physicians and occupational therapists showed most improvement, and physical therapists, psychologists, and social workers showed least improvement (the authors suggest that this could be due to the fact that psychologists and social workers were the ones with prior training on this issue, while physical therapists were one of the categories that found it most difficult to apply this knowledge to their daily job). During the follow-up period, most disciplines did not show a significant change. Only nurses showed a significant, but small, improvement.

The duration of the training was judged ‘‘good’’ by the large majority of all participants (76.5%). Immediately after the training, it was judged as ‘‘not useful’’ by 2.9%, ‘‘moderately useful’’ by 56.1%, and ‘‘very useful’’ by 40.9% of all participants, without significant differences between disciplines. At follow-up, these percentages were similar.

Possibilities to apply the lessons learned obtained varied judgements from the different disciplines, being considered positive (moderately, good, or very good) by 88.5% of the physicians, 71.4% of psychologists/social workers, 64.3% of other disciplines, 54.5% of occupational therapists, 52.9% of physical therapists, and 51.9% of the nurses. This study showed, first, that self-perceived sexological competence of rehabilitation professionals is different between disciplines. Second, this study showed that brief discipline-specific training resulted in improvement of self-perceived sexological competence in all disciplines, although some disciplines profited more than others.

In another study [18], a brief individual education session was provided. These education sessions focused on sexuality issues experienced by mental health consumers, sexual safety, sexual abuse and exploitation, sexual vulnerability, and sexual function problems associated with medication. The education session included a discussion of the BETTER model. The BETTER model was introduced as a structured approach to addressing sexual issues in oncology settings. The model comprises of six individual stages:

B = “bring up”, where the HCP raises the issue of sexuality with clients. By raising the issue, even if the client chooses not to respond, HCPs are informing that they are open and willing to discuss these issues if the client wishes to do so at a later date;

E = “explain”, where it requires HCPs to explain that for many, sexuality is an important quality-of-life issue, and that they are open to discussing these issues;

T = “tell”, where the HCP tells the client that even if an immediate solution to their concern is unavailable at this time, a referral will be made to a specialist service to assist;

T = “time”, where the HCP times the discussion to the client’s preference;

E = “educate”, where there is the need to educate clients regarding sexual side-effects of treatments;

R = “record”, the assessment, treatment, and outcome in the client’s medical record [24]. The education session took approximately 40 minutes to complete for each participant, and signified the end of their initial interview. The participants were then asked to trial the use of the model with consumers over a four-week period. The BETTER model was described by most participants as easy to understand, and they thought the structure was not complicated, which assisted them to include the approach in their practice. Because of its simplicity, participants described the approach as steadily becoming part of their practice. Most participants did not use the model exactly as presented, but rather tended to adapt it to suit their own style, to find a way to introduce the topic in a way that felt natural for them. A further study [20] found that the same intervention still had a positive outcome after two years.

In a study on attitudes and beliefs of residential aged-care staff [22], the education intervention (delivered as a three-hour workshop) was designed specifically for nursing staff. Topics covered in the first hour included the following: what sexuality is and why it is important; common attitudes towards sexuality in older people; sexual stereotypes (including nonheterosexuality) versus the reality; sexuality and normal aging; sexuality, illness, and treatment; and sexual expression in residential care. The second hour covered the following topics: sexuality and people with dementia; sexuality, dementia, assessment, and consent; sexuality and the role of residential aged-care staff; and residents’ rights and staff responsibilities. The third hour covered legal issues, including capacity and consent for residents with dementia, and was delivered in the form of a 50-minute DVD presented by a lawyer with expertise in the area of aged care [22]. A chi-square analysis showed that, overall, attitudes were significantly more permissive following the education intervention. An education intervention of relatively short duration can have a significant impact on the sexuality of older adults.

In Fronek, Booth, Kendall, Miller, and Geraghty’s [14] study, an education program, informed by a local needs assessment, was developed and delivered by an external clinical nurse consultant who is a recognized specialist in the area of sexuality and spinal cord injury (SCI). The Permission, Limited Information, Specific Suggestions, and Intensive Therapy (PLISSIT) model [25], widely accepted and utilized in sexuality education, provided a conceptual framework for training which assists individual participants with varying levels of knowledge, skill, and comfort levels to manage sexual questions at a minimum level of competency (sexual problems, infertility, case studies, sexual identity, disability, medication, surgical implants, contraception, group discussion). It provides a process framework that allows for different degrees of involvement based on the staff member’s comfort level, knowledge base, and counseling skills. This model is particularly appropriate to be used within the interdisciplinary team, where different members contribute varying levels of skill, knowledge, and experience in sex counseling. Post hoc comparisons revealed that the treatment group showed significant changes between pre- and post-education on the Knowledge (Z = −5.706, *p* < 0.001), Comfort (Z =−4.306, *p* < 0.001), Approach (Z =−4.555, *p* < 0.001), and Attitude (Z =−3.4996, *p* < 0.001) subscales. At the 3-month follow-up, the treatment group reported significantly higher scores than they did at pre-education for the subscales of Knowledge (Z = −5.116, *p* < 0.001), Comfort (Z = −3.953, *p* < 0.001), Approach (Z = −4.103, *p* < 0.001), and Attitudes (Z = −2.655, *p* < 0.01). Although scores did tend to be lower at the 3-month follow-up than immediately following the education, they did not differ statistically. In identifying and developing appropriate content for inclusion in training programs, the utility of the knowledge, comfort (general and personal), and attitude framework was taken into account [26]. By specifying these domains and identifying the current status and individual needs of staff in each of these domains, appropriate content could be developed and tailored to the team, inclusive of the psycho-emotional needs of individual staff, as well as their identified knowledge needs. Whether improvements can be maintained beyond 3 months and for how long is of theoretical and practical importance when considering the need for ‘refresher’ courses or additional training initiatives.

In order to explore the long-term benefits, this study was conducted as a two-year follow-up to a randomized controlled trial of a sexuality training program for practitioners employed in spinal cord injury rehabilitation [17]. The study aimed to determine whether reported gains in the experimental group at three months were maintained at two years and whether the control group, who received training in the fourth month, had similarly improved knowledge, comfort, and attitudes at the two-year period. As hypothesized, there were no significant between-group differences in knowledge, comfort, or attitude at two-year follow-up, indicating that both experimental and control groups had similar levels of knowledge, comfort, and attitude. This contrasts with the significant between-group differences previously reported at the 3-month follow-up, and highlights that the training of the control group at the four-month period resulted in equalisation of the groups. Although within-group changes were observed in the experimental group overall but not within the control group, post hoc comparisons in the control group revealed significant changes in knowledge and attitude between three months and the two-year follow-up. Thus, while there was no overall pattern of change for the control group, the period of post-training for this group did demonstrate significant changes in knowledge and attitude, supporting the effectiveness of training.

A one-day education programme was developed and delivered by an interdisciplinary team of staff (psychologist, nurse, care staff, physiotherapist, occupational therapist) working within the hospital where the study took place. The PLISSIT model was adopted as the conceptual framework for the development of the education day. Results from the knowledge scale indicated that there was a significant difference between the pre- and post-scores for the overall knowledge score and for the majority of items within the scale [18].

## 4. Discussion

Leiblum [27], in a study of US medical schools, found that 61% of schools provided 10 hours or less of education in human sexuality, and a mere 15% provided 20 hours or more. The findings of the analyzed studies seem to support the suggestion that educational interventions designed to improve sexual health care practices should first focus on deepening health professionals’ own awareness of possible barriers before improving knowledge and skills, and/or simply acknowledge [28] that not all health professionals can, or want to become an expert in sexual health care, and instead adopt a team approach in order to provide optimal care [29].

A study conducted in Canada found that the topics most likely to receive considerable or heavy emphasis were information and skills for contraception (97.6%), information and skills for prevention of STIs (75.6%), sexual violence/assault (73.2%), and female sexual dysfunction (73.2%). The topics least likely to receive considerable or heavy emphasis across disciplines were childhood sexuality (17.1%), sexuality and disability (22.0%), sexuality and aging in males (24.4%), and social and cultural differences in sexual beliefs and customs (26.8%). The topic “information and skills for contraception” was, by a wide margin, the sexual health topic that received the highest number (97.6%) of considerable or heavy emphasis responses. The topic “information and skills to prevent STIs other than HIV” received the next highest number of considerable or heavy emphasis responses (75.6% of all programs). In both instances, the three fields were similar in the percentages measuring considerable or heavy emphasis [30].

Literature converges on the need to train health professionals in full, avoiding delegating this issue only to specialists in the field. In fact, many pathologies involve a correlation with periods of sexual problems, and patients should be listened to in the entirety of their bio-psycho-social dimension. There is a need for training in the reception and care of the person who addresses the health system. There is still much to be done for all those health workers who find themselves unprepared to provide information and re-education on more specific issues or in facing situations that are still underestimated. Every patient, whatever pathology they suffer from, has their own sexuality. Taking this into account means offering the person a holistic view of taking charge, especially in cases where the disease may have upset his/her biopsychosocial balance. 

The results of most of the studies highlight that talking about sexual health both by patients and by professionals is still taboo, mostly because of the personal discomfort experienced [3].

All studies and research showed, therefore, that training is crucial for efficient and effective communication. However, training is really quite varied, based on the duration (we have seen that the training duration can go from 40 minutes to two years) and on the content (more lecture-based or more interactive).

If the HCP knows how to listen, he or she can offer the patient active listening as an important instrument. This will allow two levels of care: the first allows the HCP to deepen the anamnesis of the disease in its etiopathogenesis. Understanding it means re-educating the patient on attention to the self in its holistic entirety, recognizing its value, and transmitting to the person the importance of attention to mind–body integrity. The second level comes from the “assisted person”, as in feeling listened to, recognized, and identified as a person and not only as “chance”, to get back in touch with subjective experience, then mentalizing the role of their experiences, personal history, needs, and knowing how to start effective communication with the HCPs. The second level implies, as a “secondary” effect, awareness, and this is the basis for prevention of future pathologies and/or discomfort.

These two levels form the basis and consequence of the circular causality model, which sees reality as the effect of a complexity that can no longer be reduced to a reductionist vision. The language changes, and the relationship changes. This systemic vision, both relational and intrapersonal, leads also to an impact on the design of increasingly specific training courses. The trainer, in designing the course, should modulate it both in proposing the most technical and sanitary aspects (body) and in proposing the areas that relate to the internalized reality of both the operator and the target to which he will address. 

The studies conducted in recent years have shown that sexuality is a theme related to the historical period and culture [3], thus demonstrating the need to often rework the beliefs that underlie this area. To train also means to give the HCP the possibility to elaborate on the personal motivations that lead them to avoidance, or bias that translates into a subjective perspective, if not even into behavior; to broaden their anthropological and sociological culture. This also implies the always desirable possibility to have a common language among the different professionals, increasing the success rate of the therapeutic path.

The ultimate goal of this training path is that professionals can acquire theoretical knowledge (knowledge), practical knowledge (knowing how to do), and awareness (knowing how to be). The realization of these objectives requires contemporary work on the content and personal resonances of sexual issues, and therefore adequate time to elaborate on all this.

Inclusion of sexuality training for HCPs can contribute to the integration of this important issue in the clinical routine. Education may facilitate the ability of students and health care professionals to no longer assume that patients do not want to talk about sexuality, which would further lower their level of embarrassment and their proactivity in addressing the issue with their patients. Of course, the development of health care professionals who are well-equipped to discuss sexuality would enable the issue to be more frequently addressed in clinical settings. Sexuality is an important issue that needs to be highlighted, and if health care professionals truly want to deliver holistic care, then they surely need to address their clients’ sexual needs [31].

Medical training, therapeutic guidelines, and awareness among medical professionals and health care facilities all need to be intensified and oriented towards openness in their perspectives towards sexuality and active debunking of the myths and cultural conservatism that surround it. HCPs must regularly inform themselves and address the sexual problems of their patients, making sure that they do not judge and understand that thereby, many conditions are likely to be treated.

## 5. Conclusions

For many HCPs, the sex and sexuality of patients are areas which they may be reluctant to address or engage. Health care practitioners require education in the area of sexuality, regardless of their discipline. Further evaluations of interdisciplinary sexuality education programmes should use a larger sample and explore the differences across disciplines. Future researchers should consider testing the effectiveness of the programmes within the practice setting and across time [18]. This review demonstrates that an education on sexuality for students and health care professionals can have a positive and beneficial effect on knowledge and promote a positive attitude and self-efficacy related to sexual health care. It is possible to teach the subject of sexuality in frontal lectures; however, in light of the nature of the topic, teaching by case study and roleplay has considerable advantages over the more formal lecture system. The longevity of changes and their impact on care delivery and resident outcomes requires further investigation [22].

The topic of sexuality has no simple solutions that can be conceived of in terms of correctness or incorrectness. Doing so ignores the fundamental meaning of sexuality, and not addressing issues surrounding concepts such as the self, autonomy, and freedom of communication on issues of sexuality and not giving access to good-quality teaching delivered in a clear, non-judgmental fashion (as part of the idea of totality) may be a contributing factor to poor sexual health [32]. Training of health professionals and the provision of communication tools to health professionals can facilitate an effective dialogue between patients and health professionals. From this review, we can suggest the importance of doing at least a minimal formation to health care professionals, but of course, the possibility of organizing longer training sessions coupled with interactive organizations can enhance the possibility of health care professionals feeling more comfortable and secure in treating patients with sexual issues.

## Figures and Tables

**Table 1:** Authors, year of publication, objective, and targets of articles selected for the review.

Authors and Year of Publication	Objective	Target
Stokes and Mears, 2000 [13]	Evaluation of the impact of previous training in nurses on attitudes towards discussing sexual health issues with patients	Nurses in one English health district
Fronek et al., 2005 [14]	Evaluation of a training program in sexuality for a rehabilitation interdisciplinary team	Spinal cord injury (SCI) rehabilitation interdisciplinary team in Australia
Simpson et al., 2006 [15]	Evaluate the effectiveness of a staff training program in sexuality as a means of improving the rehabilitative management of clients’ sexual concerns after neurological disability	Multidisciplinary team involved in rehabilitation and disability at major rehabilitation centres in the North and South Islands of New Zealand
Post et al., 2008 [16]	Assess a sexological training for rehabilitation professionals in The Netherlands	Multidisciplinary team in two rehabilitation centers in The Netherlands
Fronek et al., 2011 [17]	Investigate whether the changes in the treatment group observed at the training 3- month-follow-up were maintained and compare to the control group	Interdisciplinary team in a spinal cord injury rehabilitation service in Australia
Higgins et al., 2012 [18]	Evaluate the effectiveness of an interdisciplinary sexuality education program for staff working with people with acquired physical disability	Interdisciplinary team in a hospital in Ireland
Quinn and Happell, 2012 [19]	Explore mental health nurses’ experiences of using the BETTER model to assist in raising the topic of sexuality with patients	Nurses in a mental health service in Australia
Sung and Lin, 2013 [20]	Evaluate the effectiveness of sexual health care education provided for nursing students in dealing with sexual issues	Senior nursing students in a Nursing School in Taiwan
Quinn et al., 2013 [21]	Investigate whether the participants continue addressing sexual issues as part of practice after using the BETTER model for 2 years	Nurses in a mental health service in Australia
Bauer et al., 2013 [22]	Evaluate an educational program delivered to residential aged care nurses to improve their knowledge and attitudes towards older people’s sexuality in this context	Residential aged care nurses and staff in two regional health service in Australia
Jonsdottir et al., 2016 [23]	Evaluation of the impact of a training on the perception of having enough knowledge and training in providing sexual health care	Oncology health care professionals in a University Hospital in Iceland
